# Epidemiological trends and age-period-cohort effects on lip and oral cavity cancer burden across the BRICS from 1992 to 2021

**DOI:** 10.3389/fonc.2025.1539417

**Published:** 2025-02-17

**Authors:** Zhengrong Yu, Yuhang Wu, Yu Cao, Peiyu Cheng

**Affiliations:** ^1^ Department of Stomatology, Zhuhai Hospital of Integrated Traditional Chinese and Western Medicine, Zhuhai, China; ^2^ Department of Epidemiology and Health Statistics, Xiangya School of Public Health, Central South University, Changsha, China; ^3^ School of Public Health, Jiangxi Provincial Key Laboratory of Preventive Medicine, Nanchang University, Nanchang, China; ^4^ Nursing Department, Xiangtan Central Hospital, Xiangtan, China

**Keywords:** lip and oral cavity cancer, incidence, time trend, age-period cohort model, BRICS

## Abstract

**Background:**

Lip and oral cavity cancer (LOC) is one of the common malignant tumors of the head and neck, posing significant health and economic burdens. The BRICS, including Brazil, Russia, India, China, and South Africa, represent a large global population, presenting unique public health challenges. This study aims to evaluate the epidemiological trends and variations in the burden of LOC across BRICS in a timely manner.

**Methods:**

Data on the number, all-age rate, age-standardized rate, and relative change in LOC incidence from 1992 to 2021 within BRICS were obtained from the Global Burden of Disease study (GBD) 2021, and we analyzed global and BRICS-specific LOC incidence trends over 30 years. Furthermore, age-period-cohort model was applied to estimate net drift, local drift, age, period and cohort effects between 1992 and 2021.

**Results:**

In 2021, the BRICS nations reported 194.74 thousand new LOC cases, constituting 46.2% of the global total. From 1992 to 2021, all BRICS countries witnessed a significant rise in LOC cases, with China leading at 259.06%. The age-standardized incidence of LOC increased by over 20% in the Russian Federation, India, and China, while Brazil and South Africa exhibited marginal changes (Brazil: 0.75%; South Africa: -7.87%). Rising LOC trends were prevalent across most age groups in China, India, and the Russian Federation, particularly affecting older adults (60-94 years). Age, period, and cohort effects were deteriorating in China and India, contrasting with improvements in Brazil and South Africa.

**Conclusion:**

LOC incidence has increased across BRICS, with temporal trends not consistently aligning with economic growth and exhibiting significant variation among countries. Brazil’s experience highlights the efficacy of oral health and tobacco control measures in mitigating LOC, especially in fast-developing nations. Prevention should target men and elderly in China and India, and women in other areas.

## Introduction

Lip and oral cavity cancer (LOC) is a type of squamous cell carcinoma of the head and neck, which is one of the common malignant tumors of the head and neck, usually occurring on the lips, tongue, gums, floor of the mouth, upper and lower jaws (1). The Global Burden of Disease (GBD) 2019 study indicates that the number of Disability-Adjusted Life Years (DALYs) attributable to lip and oral cancer reached 5.51 million person-years in 2019, ranking 13th in the global malignant tumor burden of disease parity, resulting in an estimated 370,000 new cases and 199,000 deaths in 2019 (2, 3). Although the mortality and disease burden of LOC are not high in the total cancer population, the quality of life of those who survive is significantly reduced due to the severe impact on eating (4). Furthermore, there is a notable geographical variation in the incidence of oral cancer, with the situation being particularly critical in low- and middle-income countries in comparison to high-income countries (5).

Brazil, Russian Federation, India, China and South Africa (BRICS) were grouped together due to their fast-growing economies and their collective population accounting for nearly half of the global total (6). All BRICS countries are high-burden countries for LOC disease, with DALYs in BRICS countries exceeding half of the global LOC burden (7). Previous studies have tracked the global LOC burden and assessed global LOC incidence trends over time, but few studies have analyzed LOC incidence trends in the BRICS countries (3, 8). Studies on the incidence of LOC have been conducted in Brazil and China, providing information on the incidence of LOC in the BRICS countries (9–11). However, these studies were not exhaustive in terms of the countries included and comparisons between BRICS countries were not made. Given the widely recognized link between economic prosperity and improved health widely recognized, many policymakers view the BRICS countries as pivotal for altering global health as their economies grow rapidly (12). It is therefore of great importance to regularly assess, update and compare LOC incidence within the BRICS countries and to evaluate their progress in LOC prevention and treatment. The BRICS countries and other high-burden countries require this information to inform the allocation and strategic adjustment of healthcare resources.

The aim of this study was to analyze the incidence of LOC in the BRICS countries from 1992 to 2021, visualized by age, sex and year, providing an up-to-date epidemiological overview of LOC in the BRICS countries. Subsequently, the impact of age, period and birth cohort on changes in LOC incidence over the past 30 years was further assessed using the age-period-cohort (APC) model. The findings of this study therefore represent a significant addition to the existing evidence on the burden of LOC in the BRICS countries, providing valuable insights into healthcare resource allocation and future health policy formulation.

## Method

### Data sources

The data used in this study were obtained from GBD 2021 public dataset, which can be accessed at the Global Health Data Exchange GBD Results Tool (https://ghdx.healthdata.org/gbd-2021). GBD 2021 provides multilevel data on the burden of disease for 371 diseases and injuries in 204 countries and territories around the world, covering the most comprehensive burden of disease, risk, death and disease-related disability on health, and is the most important database for understanding the global burden of disease (7, 13, 14). GBD 2021 includes several significant updates: 19,189 new DALY data sources, 12 new causes, and various methodological improvements. Additionally, it incorporates the impact of the COVID-19 pandemic on the burden of disease (7).

In GBD 2021, LOC is defined according to the International Classification of Diseases, 9th edition (ICD-9): 140-145.9, 210.0-210.6, 235.0 and 10th edition (ICD-10): C00-C08.9, D10.0-D10.5, D11-D11.9 (14). In this study, we extracted estimates of LOC incidence from GBD 2021 by sex (female, male, and both), age (0-94 years), and year (since 1992) for the BRICS countries from 1992 to 2021. The 95% uncertainty intervals (UIs) were obtained by replicating the sample 1000 times, with upper and lower bounds determined by the 2.5th and 97.5th percentiles of the uncertainty distribution (7). Methodological information and details of the modelling strategy for GBD 2021 have been published elsewhere (7, 13, 14). The relevant data were anonymized and publicly available, and the informed consent waiver was reviewed and approved by the University of Washington Institutional Review Board.

### Statistical analysis

#### Descriptive analysis of LOC incidence

This study provides a statistical description of the incidence characteristics of LOC in the BRICS countries from 1992 to 2021. It examines the epidemiological characteristics and trends of LOC within the BRICS countries by analyzing the incidence across different sex and age groups.

#### Age−period−cohort modelling analysis of incidence data

The APC modeling is a common statistical tool for extracting information hidden in epidemiological results. It analyzes disease risk and explores influencing factors in terms of age, period and cohort dimensions to extract and reveal possible information about disease patterns (15, 16). In this study, the age-period-cohort model with Intrinsic Estimator (IE) method was used to assess the independent effects of age, period and birth cohort on LOC incidence. Age, period, and cohort effects were calculated via Poisson regression, expressed as follows:


$$ɡ(Y_{j}/\text{μ})=\text{log}(\lambda_{j})=u+\alpha \;aɡe_{j}+\beta \;period_{j}+\gamma \;cohort_{j}$$

where $\lambda_{j}$ represents the response variable of the net effect on LOC incidence for group j ; $Y_{j}$ and μ represent the number of incidences and the population at risk, respectively. α , β , and γ represent the coefficients of age, period, and birth cohort of the APC model, respectively. u represents the intercept of the model.

In the APC model, the results of the model provide several important indicators: net drift, local drift, longitudinal age curve, period relative risk (RR) and cohort relative risk. Net drift, which represents the log-linear trend by period and cohort for the whole population; local drift, which represents the log-linear trend by period and cohort for each age group; longitudinal age curve, which represent the expected age-specific rates in the reference cohort adjusted for period effects; and period (or cohort) RR, which represents the RR of the population across different periods (or cohorts) adjusted for both age and cohort (or period) (17).

The APC model required that the age group data and the period group data be structured in the same manner. We therefore grouped the LOC incidence, population data according to the following rules. Age group: the total population was divided into 16 age groups at 5-year intervals: 15-19 years to 90-94 years. Period group: This was divided into 6 consecutive 5-year periods, from 1992-1996 (median 1994) to 2017-2021 (median 2019). Birth cohort group: birth cohort was calculated as period - age, divided into 21 consecutive birth cohorts (from 1900-1904 to 2000-2004). In the APC model of this study, the period (2002-2006) and the birth cohort (1950-1954) were used as reference groups for calculating the RR. When the RR value is greater (less) than 1, it indicates that the risk of LOC incidence is increased (decreased). We obtained estimated parameters using APC analyses with the age-period-cohort web tool designed by the National Cancer Institute and plotted using the R statistical program (version 4.2.3). To test the significance of evaluable parameters and functions, the Wald χ^2^ test was used, and all statistical tests were two-sided.

The study is reported according to Strengthening the Reporting of Observational Studies in Epidemiology criteria, and a checklist is provided as online appendix material.

## Result

### Incidence of lip and oral cavity cancer trends from 1992 to 2021

**Table 1** shows the population, total incidence, all-age incidence rate, age-standardized incidence rate, and net drift of incidence for the world and BRICS countries. In 2021, there were 421.58 thousand (95% UI 389.88, 449.78) global LOC cases, with BRICS countries accounting for 194.74 thousand cases, or 46.2% of the total. Over the past 30 years, LOC incidence has increased significantly in all BRICS countries, with China experiencing the highest growth at 259.06%. India led in LOC cases with 112.45 thousand (95% UI 96.27, 126.07) cases.

**Table 1:** Trends in lip and oral cavity cancer incidence across BRICS, 1992-2021.

Characteristics	Global			Brazil			Russia Federation			India			China			South Africa		
	1992	2021	Change^†^, %	1992	2021	Change^†^, %	1992	2021	Change^†^, %	1992	2021	Change^†^, %	1992	2021	Change^†^, %	1992	2021	Change^†^, %
Population (Number^*^, n x 1,000,000)																		
Both	5497 (5379, 5624)	7891 (7667, 8131)	43.55	153 (142, 165)	220 (188, 251)	43.74	152 (138, 166)	145 (125, 164)	-4.54	885 (819, 951)	1414 (1240, 1602)	59.78	1206 (1111, 1302)	1423 (1319, 1530)	17.95	39 (35, 42)	57 (50, 64)	47.03
Female	2727 (2670, 2788)	3932 (3823, 4049)	44.18	78 (72, 84)	113 (96, 128)	45.33	81 (73, 88)	77 (67, 87)	-3.91	424 (393, 456)	690 (605, 782)	62.62	583 (537, 629)	695 (644, 747)	19.15	20 (18, 22)	29 (25, 33)	45.29
Male	2770 (2709, 2835)	3959 (3844, 4082)	42.93	76 (70, 81)	108 (92, 122)	42.11	71 (65, 78)	67 (58, 76)	-5.26	461 (426, 495)	724 (635, 820)	57.16	623 (574, 673)	728 (675, 783)	16.83	19 (17, 20)	28 (24, 32)	48.88
Incidence (Number^*^, n)																		
Both	184370 (176898, 191726)	421578 (389879, 449783)	128.66	3741 (3546, 3928)	9648 (8999, 10255)	157.91	9795 (9541, 10057)	14306 (12952, 15560)	46.05	37679 (33797, 41763)	112448 (96270, 126069)	198.43	15696 (13718, 17695)	56360 (45179, 69805)	259.06	1030 (802, 1227)	1974 (1720, 2209)	91.64
Female	59462 (55574, 63009)	148660 (135704, 160404)	150.01	901 (847, 943)	2740 (2504, 2903)	204.08	2003 (1917, 2069)	4059 (3617, 4487)	102.63	13059 (10860, 15414)	41782 (35240, 48590)	219.94	5584 (4676, 6579)	14711 (11411, 18444)	163.45	282 (233, 342)	616 (546, 690)	118.30
Male	124908 (119208, 130660)	272917 (245321, 296016)	118.49	2840 (2677, 3004)	6908 (6411, 7469)	143.27	7792 (7565, 8045)	10246 (8998, 11307)	31.50	24620 (21514, 27999)	70665 (55690, 83630)	187.02	10112 (8432, 11966)	41648 (31155, 54224)	311.85	748 (552, 917)	1358 (1140, 1535)	81.59
All-age incidence rate (Rate per 100,000^*^)																		
Both	3.35 (3.22, 3.49)	5.34 (4.94, 5.70)	59.29	2.44 (2.31, 2.56)	4.38 (4.08, 4.65)	79.43	6.46 (6.29, 6.63)	9.88 (8.94, 10.74)	53.00	4.26 (3.82, 4.72)	7.95 (6.81, 8.91)	86.78	1.30 (1.14, 1.47)	3.96 (3.18, 4.91)	204.42	2.66 (2.07, 3.17)	3.47 (3.02, 3.88)	30.34
Female	2.18 (2.04, 2.31)	3.78 (3.45, 4.08)	73.40	1.16 (1.09, 1.22)	2.43 (2.22, 2.57)	109.24	2.49 (2.38, 2.57)	5.24 (4.67, 5.80)	110.87	3.08 (2.56, 3.63)	6.05 (5.11, 7.04)	96.74	0.96 (0.80, 1.13)	2.12 (1.64, 2.66)	121.11	1.41 (1.17, 1.71)	2.12 (1.88, 2.38)	50.25
Male	4.51 (4.30, 4.72)	6.89 (6.20, 7.48)	52.87	3.75 (3.54, 3.97)	6.42 (5.96, 6.94)	71.18	10.94 (10.63, 11.30)	15.19 (13.34, 16.76)	38.80	5.34 (4.67, 6.08)	9.76 (7.69, 11.55)	82.63	1.62 (1.35, 1.92)	5.72 (4.28, 7.45)	252.53	4.00 (2.95, 4.90)	4.87 (4.09, 5.51)	21.97
Age-standardized Incidence rate (Rate per 100,000^*^)																		
Both	4.32 (4.14, 4.50)	4.88 (4.52, 5.20)	12.89	3.76 (3.55, 3.95)	3.78 (3.52, 4,02)	0.75	5.31 (5.17, 5.45)	6.43 (5.82, 6.98)	21.07	6.93 (6.17, 7.77)	9.02. (7.77, 10.12)	30.11	1.73 (1.52, 1.95)	2.68 (2.15, 3.30)	54.77	4.36 (3.55, 5.24)	4.02 (3.52, 4.48)	-7.87
Female	2.65 (2.47, 2.81)	3.28 (3.00, 3.54)	23.78	1.78 (1.67, 1.87)	1.98 (1.82, 2.10)	11.35	1.76 (1.68, 1.81)	3.09 (2.75, 3.42)	75.97	5.01 (4.13, 6.02)	6.64 (5.59, 7.75)	32.48	1.21 (1.02, 1.42)	1.38 (1.07, 1.73)	13.83	2.09 (1.70, 2.56)	2.26 (2.00, 2.53)	8.32
Male	6.21 (5.93, 6.49)	6.65 (5.99, 7.21)	7.20	5.97 (5.61, 6.31)	5.89 (5.46, 6.37)	-1.23	10.36 (10.06, 10.71)	10.80 (9.50, 11.89)	4.18	8.73 (7.61, 9.98)	11.49 (9.15, 13.56)	31.69	2.32 (1.95, 2.72)	4.13 (3.12, 5.32)	77.87	7.36 (5.34, 9.09)	6.34 (5.41, 7.12)	-13.82
APC model estimates (Net drift of incidence rate^#^, % per year)																		
Both	0.51 (0.45, 0.57)			-0.11 (-0.28, 0.59)			0.42 (0.12, 0.72)			0.57 (0.43, 0.71)			1.94 (1.81, 2.08)			-0.71 (-1.06, -0.37)		
Female	0.76 (0.71, 0.81)			0.32 (0.02, 0.61)			2.13 (1.86, 2.40)			0.49 (0.29, 0.68)			0.42 (0.30, 0.55)			0.04 (-0.53, 0.62)		
Male	0.38 (0.30, 0.45)			-0.23 (-0.45, -0.01)			-0.16 (-0.54, 0.22)			0.69 (0.56, 0.82)			2.73 (2.55, 2.91)			-0.92 (-1.37, -0.47)		

All-age incidence=crude incidence rate.

Age-standardized incidence rate is computed by direct standardization with global standard population in GBD 2021.

Net drifts are estimates derived from the age-period-cohort model and denotes overall annual percentage change in incidence, which captures the contribution of the effects from calendar time and successive birth cohorts.

APC, age-period-cohort.

*Parentheses for all GBD health estimate indicate 95% uncertainty intervals due to the inherent characteristics of model selection, parameter estimation, and the quality and availability of data inputs for GBD 2021.

^#^Parentheses for net drift indicate 95% confidence intervals.

^†^Percent change of values, 1992-2021 was calculated by [value (2021)-value (1992)]/value (1992) * 100.

From 1992 to 2021, the global all-age LOC incidence rate rose from 3.35 (95% UI 3.22, 3.49) per 100,000 to 5.34 (95% UI 4.94, 5.70) per 100,000, a 59.29% increase. All BRICS countries, excluding South Africa and Russia Federation, experienced incidence rate increases above the global average, with China’s rate showing the highest increase at 204.42%. Despite this high growth, China’s incidence rate did not lead; Russian Federation had the highest incidence rate in 2021 at 9.88 (95% UI 8.94, 10.74) per 100,000. Over the past 30 years, the global age-standardized incidence rate increased by 12.89%. In the BRICS countries, Russian Federation, India, and China saw higher increases in age-standardized rates than the global average, while South Africa experienced a decrease of 7.87%. The APC model’s net drift estimates also indicated rising incidence rates in Russian Federation, India, and China, with China having the highest annual increase at 1.94% (95% CI 1.81%, 2.08%).

### Time trends in lip and oral cavity cancer incidence across different age groups

**Figure 1:**
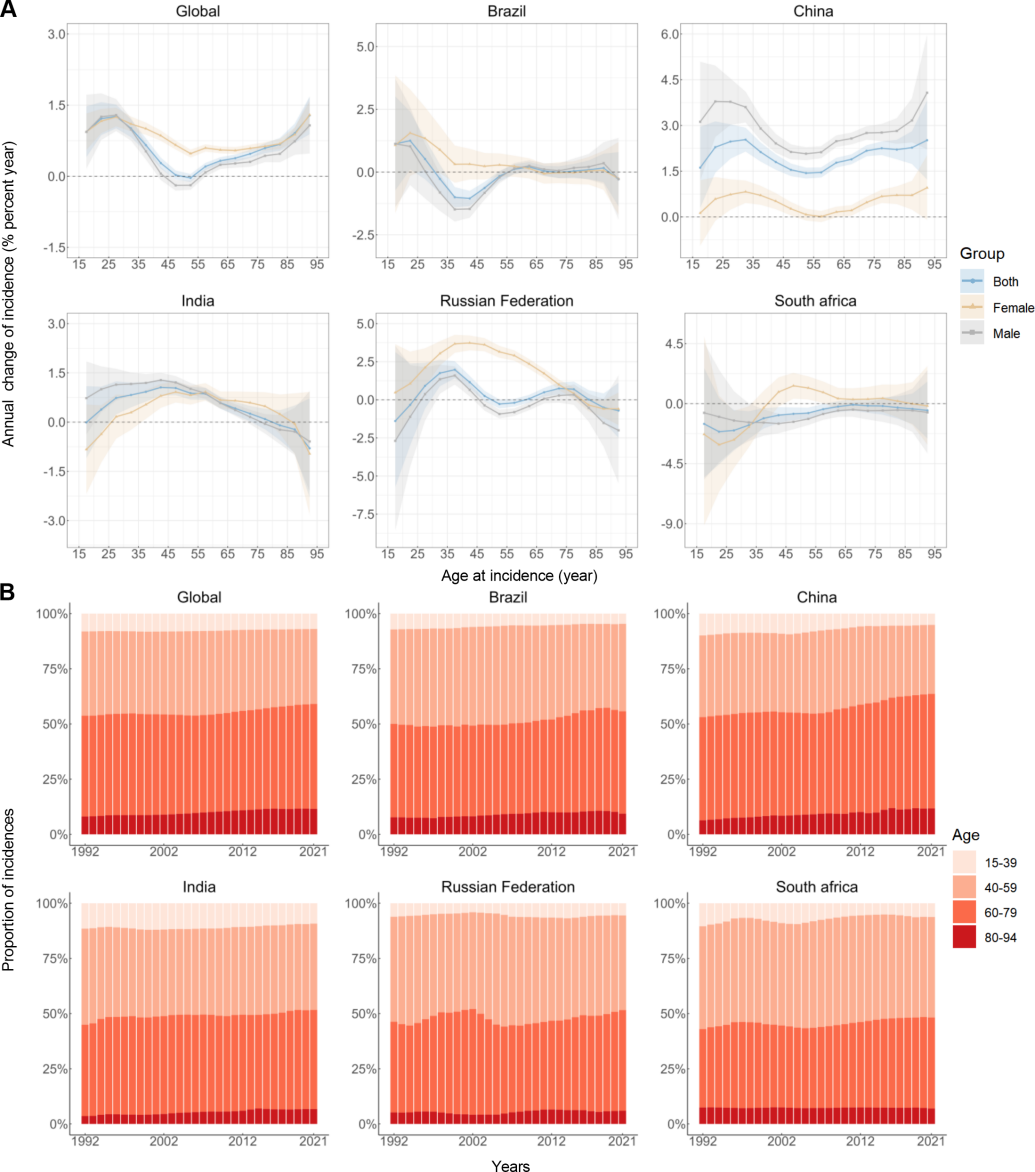
Local drifts of incidence rate and age distribution of incidences in global and BRICS, 1992–2021. **(A)** Local drifts of lip and oral cavity cancer incidence rate (estimates from age-period-cohort models) for 16 age groups (15-19 to 90-94 years), 1992–2021. The dots and shaded areas indicate the annual percentage change of incidence rate (% per year) and the corresponding 95% CIs. **(B)** Temporal change in the relative proportion of lip and oral cavity cancer incidences across age groups (15-39, 40-59, 60-79, 80-94 years), 1992–2021.

**Figure 1A** shows the annual percentage change in LOC incidence rates for each 5-year age group from 15 to 94 years. Globally, most age groups have local drift values above 0, with females generally having higher local drift values than males. Among the BRICS countries, China, India, and Russian Federation show positive local drift values for most age groups, indicating increasing LOC incidence rates. In South Africa, nearly all age groups have negative local drift values, suggesting a decline in incidence rates. Regarding gender differences, females in Brazil and Russian Federation typically have higher local drift values than males. In China, males have consistently higher local drift values across all age groups. In India and South Africa, males have higher local drift values in younger age groups, while females have higher values in older age groups.

**Figure 1B** illustrates the temporal trends in LOC cases by age group. In 1992, 53.73% of global LOC cases occurred in individuals aged 60 and above; by 2021, this proportion increased to 59.05%. A similar pattern of increasing LOC cases among the elderly (60-94 years) was observed in all BRICS countries, with China showing the highest increase from 53.09% to 63.61%. Additionally, except for South Africa, the main affected age group in these countries has shifted from middle-aged to younger elderly individuals (60-79 years). Between 2019 and 2021, the proportion of LOC cases in the very elderly population decreased in Brazil, China, and South Africa.

### Age, period and cohort effects on lip and oral cavity cancer incidence

**Figure 2:**
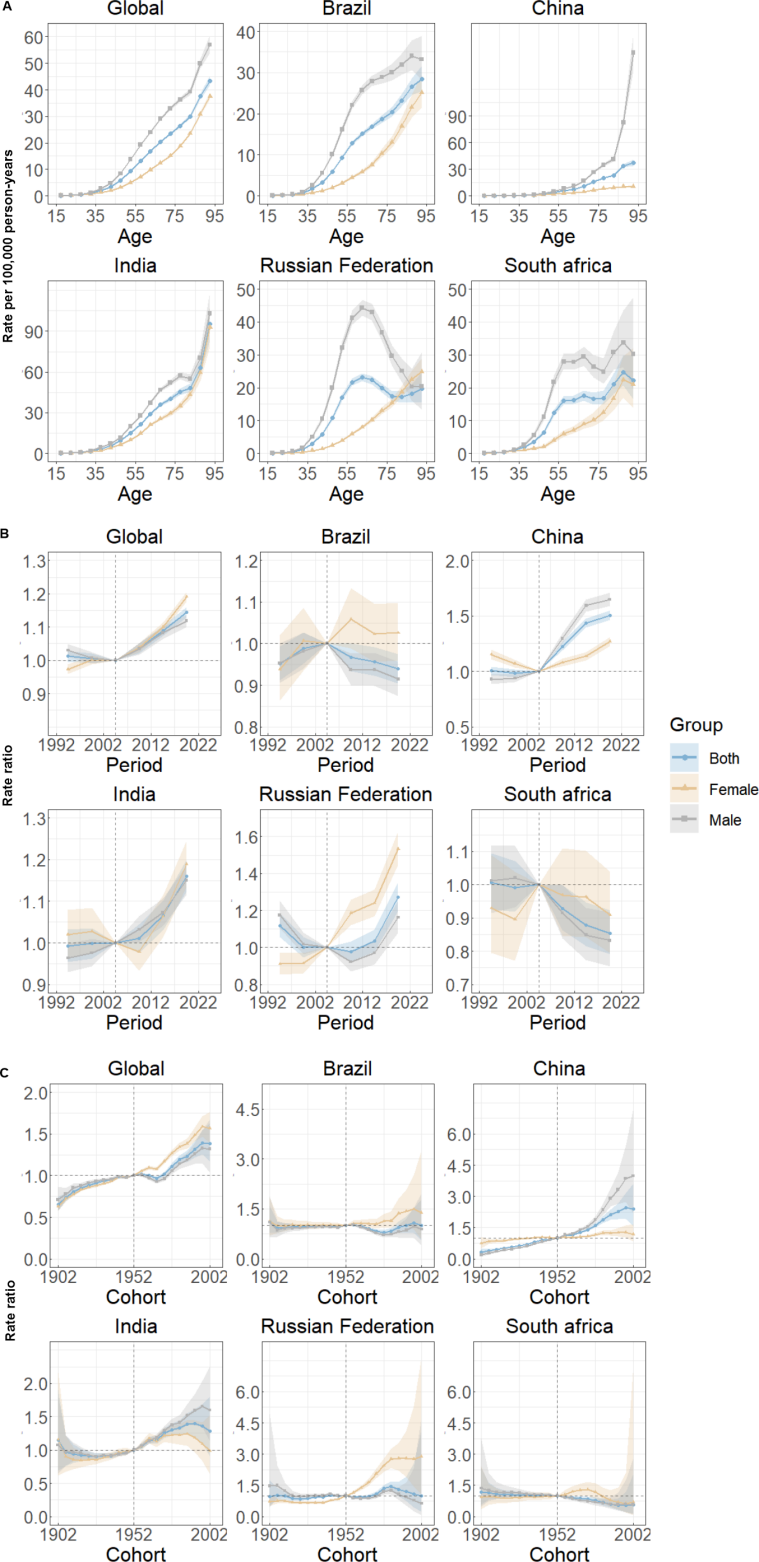
Age, period and cohort effects on lip and oral cavity cancer incidence in global and BRICS. **(A)** Age effects are shown by the fitted longitudinal age curves of incidence rate (per 100,000 person-years) adjusted for period deviations. **(B)** Period effects are shown by the relative risk of incidence rate (incidence rate ratio) and computed as the ratio of age-specific rates from 1992–1996 to 2017–2021, with the referent cohort set at 2002–2005. **(C)** Cohort effects are shown by the relative risk of incidence rate and computed as the ratio of age-specific rates from the 1902 cohort to the 2002 cohort, with the referent cohort set at 1952. The dots and shaded areas denote incidence rates or rate ratios and their corresponding 95% CIs.

**Figure 2** presents the estimated age, period, and cohort effects for the world and BRICS countries. Overall, a similar age effect pattern is observed in all countries except Russian Federation, with risk increasing as age increases. Notably, China’s male incidence rates rise sharply in old age. In Russian Federation, male risk decreases rapidly after age 60-64, while female risk continues to rise, unlike in other countries. Gender differences in age effects are seen in all BRICS countries, with males having higher risks than females (**Figure 2A**).

Over the past 30 years, the period effects for the world, China, and India show a similar “J” shape, with risk increasing and remaining above 0 after 2004. In Brazil and South Africa, period effects continuously decline, indicating effective control of LOC incidence rates over time. In Russian Federation, period effects show a “U” shape, with risk decreasing initially and then rising. Gender differences in period effects are evident, with distinct regional characteristics (**Figure 2B**).

Cohort effects over the past 30 years show that for the world, China, and India, the risk increases for individuals born after 1900. In Brazil, Russian Federation, and South Africa, cohort effects remain relatively stable, with a slight decline in South Africa for cohorts born after 1950-1954. Gender differences in cohort effects are also present, with China and India showing similar patterns, and Brazil, Russian Federation, and South Africa exhibiting another pattern (**Figure 2C**).

## Discussion

LOC is a low mortality cancer with a high survival rate if detected and treated early. However, surgery as the primary treatment modality leads to changes in facial morphology and difficulties in chewing and swallowing, which contributes to some extent to the disability burden of patients with LOC (1). Controlling LOC incidence is crucial for reducing its disease burden. Over the past three decades, despite significant advances in diagnosis and treatment of LOC (18, 19), global incidence and age-standardized incidence rates have continued to rise.

A marked heterogeneity exists in the incidence and long-term trends of LOC across the BRICS nations. Notably, the Russian Federation, India, and China each witnessed a surge exceeding 50% in the crude incidence of LOC between 1992 and 2021. Upon adjusting for variations in age distribution, the age-standardized incidence of LOC in these countries has still escalated by over 20% during the past three decades. This persistent increase implies the presence of additional, fundamental factors that are driving the observed rise in LOC incidence, beyond the influence of demographic shifts alone. China and India have experienced a significant increase in population over the past three decades (20), and this demographic expansion has been a significant contributing factor to the rise in the number of local LOC incidents. Local drift indicates that the incidence of LOC is increasing at a faster rate in all age groups in China, specifically among those aged 25-59 in India, and 25-45 in Russian Federation. Currently, both China and the Russian Federation are grappling with rapid population aging, contrasting with India’s youthful demographic profile (20). The combination of demographic changes and high risk of incidence in specific age groups will result in continued growth in the incidents and incidence of LOC in China and India. Between 1992 and 2021, LOC age-standardized incidence in Brazil and South Africa did not change much (Brazil: 0.53%, South Africa: -7.80%), but crude incidence increased. Local drift suggests that the incidence in most age groups has not maintained its growth and has even declined. The risk of LOC varies significantly among the BRICS countries, reflecting the influence of cultural practices, socioeconomic conditions, and healthcare systems. Tobacco use, alcohol consumption, betel nut chewing, and Human papillomavirus (HPV) infection are well-recognized risk factors for LOC (21), though their relative impact differs across regions due to variations in lifestyle, public health policies, and access to healthcare services. To further understand the factors influencing LOC incidence in these regions, we examined age, period, and cohort effects.

In Brazil, the age effect contributed to an increase in oral cancer incidence, while the period and cohort effects demonstrated positive trends, reflecting the country’s commendable progress in healthcare improvements and effective control of key risk factors. The National Oral Health Policy (PNSB) in Brazil prioritizes prevention and early diagnosis of oral cancer. Since its implementation in 2004, access and continuity of oral health services have improved through the expansion of Primary Health Care, the use of oral health teams (OHT) in the Family Health Strategy, and the establishment of Specialized Dental Care Centers for early diagnosis and treatment of LOC patients (22, 23). Tobacco exposure is the largest and recognized contributor to LOC (24). A 2019 report by the World Health Organization (WHO) highlights Brazil’s success in tobacco control under the guidance of the Framework Convention on Tobacco Control (25). Brazil has benefited greatly from PNSB programs and tobacco restrictions.

The decline in period and cohort effects in South Africa may reflect advancements in local tobacco regulation and lifestyle changes. Since 1993, South Africa has implemented strict tobacco control policies, including bans on tobacco advertising, restrictions on public smoking, increases in cigarette excise taxes, and health education programs (26). These measures have significantly reduced cigarette consumption, with a 54% decrease in per capita cigarette consumption recorded between 1999 and 2011 (27). In addition, efforts to manage South Africa’s high prevalence of HIV have indirectly strengthened the healthcare system, enabling the development of cancer prevention strategies. However, the healthcare system and public health response to LOC remain weak, as the primary focus has been on HIV prevention and treatment. The high prevalence of HPV—affecting more than 17% of women—and inadequate vaccination coverage exacerbate the risk of HPV-associated oral cancers, particularly in women (28). This may explain the higher risk of developing LOC in women compared to men. Looking ahead, increased investment in cancer prevention strategies and improved efforts to prevent HPV infection, such as expanding vaccination programs, are critical areas where South Africa can focus to reduce the burden of LOC.

In China and India, age, period and cohort effects all contributed to the increase in incidence, with China experiencing the highest incidence increase ratio among the BRICS countries (204.62%) and India the second highest (86.78%). The age effect suggests that in both countries, the risk of LOC incidence is highest in the elderly age groups (>80 years), much higher than in the other BRICS countries. Physical and cognitive decline in the elderly population can lead to difficulties in routine oral care and reduced access to oral care services, which in turn may exacerbate the risk of LOC incidence (29). A systematic review demonstrated that socio-economic inequality is associated with the risk of oral cancer (30). Elderly individuals in the older age groups in China and India often experience extreme poverty, which may explain the high incidence of LOC in these regions. Current dental care systems are costly and fundamentally inadequate for low-income countries to address the ongoing challenges. Countries facing these issues should emphasize the importance of educating populations on proper hygiene practices, which could yield significant public health benefits in the future. Period and cohort effects in both China and India exhibit a ‘J’-shaped trend, indicating a continued rise in the risk of developing LOC. Although a range of public health interventions have been initiated locally, including, tobacco control, limiting alcohol consumption and publicizing the human harms of betel nut chewing, the sustained effectiveness of these measures may not yet be fully realized. Anti-tobacco initiatives have faced challenges in coverage and implementation, with lifestyle inertia posing a significant barrier to achieving long-term gains. For instance, China, which accounts for over 44% of global tobacco consumption, has yet to implement a national smoke-free law (31). In India, male smoking rates tripled between 1998 and 2010 (32). Additionally, betel nut—a World Health Organization classified Group 1 carcinogen—is widely consumed in both countries, leading to severe health consequences (33). Men face a higher risk of developing LOC compared to women, largely due to sociocultural factors. Men are more likely to engage in smoking, alcohol consumption, and betel nut chewing, behaviors that significantly elevate their LOC risk. These findings align with previous research (34).

The Russian Federation was the only country where the age effect was reversed, although only in the male cohort. The period effect and the cohort effect suggested a higher risk of incidence in women. The decline in the age effect for men after 60-64 could be linked to their lower life expectancy. In Russian Federation, where men’s life expectancy rarely exceeds 64 years (14), those who have experienced LOC exposure may have died prematurely. The high risk of LOC development in women has been observed in other studies (35), and the age-standardized increase in smoking prevalence in women may be important, with smoking prevalence in middle-aged women consistently increasing, while smoking prevalence in younger women has been trending downwards, which may explain the characteristics of the female period and cohort effect.

Despite rapid economic growth in the BRICS countries, the period and pace of development vary, leading to differing trends in LOC incidence. Moreover, unique cultural practices and health policies across these nations contribute to region-specific risk factors and areas requiring intervention. However, it is important to acknowledge that successful strategies implemented in one country can offer valuable insights for other regions in reducing the burden of LOC. Brazil’s experience highlights significant gains through the implementation of PNSB and tobacco control, serving as a model for other countries. The cases of South Africa, China, India, and Russia Federation indicate the need for targeted strategies for high-risk groups within the LOC population. The World Health Assembly 2021 resolution on oral health promotes developing a global strategy to achieve universal oral health coverage by 2030 (3). Positive health policy and public health actions may significantly improve LOC prevention and treatment outcomes globally.

To the best of our knowledge, this is the first study to investigate time trends in LOC incidence in the BRICS countries using the latest GBD 2021 data, providing comprehensive coverage of periods and populations. This study also uniquely applies an APC model to examine trends, allowing us to capture important shifts in specific populations and provide targeted recommendations. This study also has some limitations, and the availability and quality of raw data is a limiting factor in estimating LOC incidence data. When high-quality or accessible LOC data from national or local cancer registries were not available, there was some discrepancy in the GLOBOCAN and GBD estimates of LOC (36), although this discrepancy in estimates was not large in the BRICS countries, which underscores the importance of expanding cancer and vital registration systems. Additionally, the GBD study did not stratify LOC, which included various anatomical site cancers with differing pathophysiological determinants, such as cancers of the lip and other parts of the oral cavity. Future studies could improve accuracy by separating LOC into specific categories based on anatomical sites.

## Conclusion

Between 1992 and 2021, the incidence of LOC has risen in the BRICS nations, with trends not aligning proportionally with socioeconomic advancement. Despite shared economic growth, regional variability in LOC incidence persists. Brazil’s experience suggests that implementing oral health programs and strong tobacco control measures can mitigate LOC incidence. Rapidly developing economies can benefit from similar efforts. Preventive strategies must prioritize at-risk populations, emphasizing men and the elderly in China and India, and increasing vigilance for women’s susceptibility in other countries.

## Data Availability

Publicly available datasets were analyzed in this study. The datasets generated during and/or analyzed during the current study are available in the GBD Data Tool repository (http://ghdx.healthdata.org/gbd-results-tool). This public link to the database of GBD study is open, and the use of data does not require additional consent from IHME.
